# Bis(cyclo­hexyl­ammonium) tetra­bromido­cuprate(II)

**DOI:** 10.1107/S1600536812011117

**Published:** 2012-03-21

**Authors:** Meng Ting Han

**Affiliations:** a Ordered Matter Science Research Center, College of Chemistry and Chemical Engineering, Southeast University, Nanjing 211189, People’s Republic of China

## Abstract

The structure of the title salt, (C_6_H_14_N)_2_[CuBr_4_], is built up from cyclo­hexyl­ammonium cations and tetra­bromidocuprate anions, the latter being located on an inversion center. In the crystal, anions and cations are inter­connected by N—H⋯Br hydrogen bonds, forming ribbons parallel to [0-11].

## Related literature

For background to the development of ferroelectric pure organic or inorganic compounds, see: Haertling (1999 ▶); Homes *et al.* (2001 ▶). For the synthesis of a variety of compounds with potential piezoelectric and ferroelectric properties, see: Fu *et al.* (2009 ▶); Hang *et al.* (2009 ▶). For the synthesis of the title compound, see: Willett (2004 ▶).
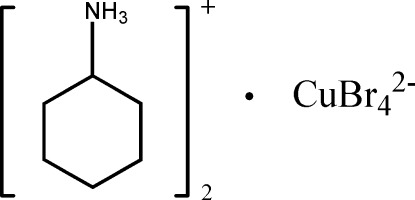


## Experimental

### 

#### Crystal data

(C_6_H_14_N)_2_[CuBr_4_]
*M* *_r_* = 583.51
Monoclinic, 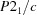

*a* = 14.372 (3) Å
*b* = 7.6483 (15) Å
*c* = 9.1561 (18) Å
β = 106.89 (3)°
*V* = 963.0 (3) Å^3^
*Z* = 2
Mo *K*α radiation
μ = 9.42 mm^−1^
*T* = 293 K
0.33 × 0.28 × 0.20 mm

#### Data collection

Rigaku SCXmini diffractometer
Absorption correction: multi-scan (*CrystalClear*; Rigaku, 2005 ▶) *T*_min_ = 0.056, *T*_max_ = 0.152
9579 measured reflections
2199 independent reflections
1468 reflections with *I* > 2σ(*I*)
*R*_int_ = 0.092
2 standard reflections every 150 reflections intensity decay: none

#### Refinement

*R*[*F* ^2^ > 2σ(*F* ^2^)] = 0.047
*wR*(*F* ^2^) = 0.095
*S* = 1.03
2199 reflections
88 parameters
H-atom parameters constrained
Δρ_max_ = 0.62 e Å^−3^
Δρ_min_ = −0.77 e Å^−3^

### 

Data collection: *CrystalClear* (Rigaku, 2005 ▶); cell refinement: *CrystalClear*; data reduction: *CrystalClear*; program(s) used to solve structure: *SHELXS97* (Sheldrick, 2008 ▶); program(s) used to refine structure: *SHELXL97* (Sheldrick, 2008 ▶); molecular graphics: *DIAMOND* (Brandenburg, 1999 ▶); software used to prepare material for publication: *SHELXL97*.

## Supplementary Material

Crystal structure: contains datablock(s) I, global. DOI: 10.1107/S1600536812011117/bh2418sup1.cif

Structure factors: contains datablock(s) I. DOI: 10.1107/S1600536812011117/bh2418Isup2.hkl

Additional supplementary materials:  crystallographic information; 3D view; checkCIF report

## Figures and Tables

**Table 1:** Hydrogen-bond geometry (Å, °)

*D*—H⋯*A*	*D*—H	H⋯*A*	*D*⋯*A*	*D*—H⋯*A*
N1—H1*D*⋯Br2^i^	0.89	2.60	3.474 (4)	166
N1—H1*C*⋯Br2^ii^	0.89	2.56	3.445 (4)	174
N1—H1*E*⋯Br3^iii^	0.89	2.62	3.341 (4)	138

Symmetry codes: (i) 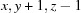; (ii) 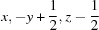; (iii) 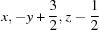.

**Fig. 1.**
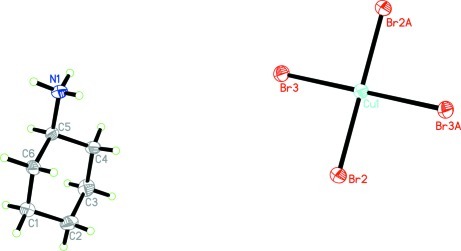
Perspective structure of the title compound, with displacement ellipsoids drawn at the 30% probability level.

**Fig. 2.**
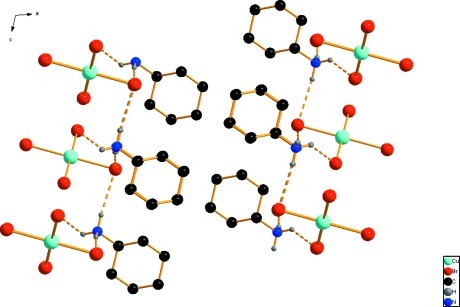
The crystal packing of the title compound viewed along the b axis showing the hydrogen bonds network. Some of the H atoms have been omitted for clarity.

## supplementary crystallographic information

### Comment

At present, much attention in ferroelectric material field is focused on developing ferroelectric pure organic or inorganic compounds (Haertling, 1999; Homes *et al.* 2001). Recently we have reported the synthesis of a variety of compounds (Fu *et al.*, 2009; Hang *et al.*, 2009), which have potential piezoelectric and ferroelectric properties. In order to find more dielectric ferroelectric materials, we investigated the physical properties of the title compound (Fig. 1). The dielectric constant of the title compound as a function of temperature indicates that the permittivity is basically temperature-independent (dielectric constant equaling to 0.42 to 6.6), suggesting that this compound should be not a real ferroelectrics or that no phase transition occurs within the measured temperature range. Similarly, below the melting point (443 K) of the compound, the dielectric constant as a function of temperature also goes smoothly, and there is no dielectric anomaly observed. Herein, we report the synthesis and crystal structure of the title compound.

The structure of the title compound is shown in Fig. 1. There are one half CuBr_4_^2-^ anion and one cyclohexylammonium cation in the asymmetric unit. The cyclohexyl ring has the chair conformation. As it can be seen from the packing diagram (Fig. 2), ions are connected *via* intermolecular N—H···Br hydrogen bonds to form a one dimensional chain in the crystal. Dipole–dipole and van der Waals interactions are effective in the molecular packing.

### Experimental

Crystals of the title compound were grown by evaporation of an aqueous solution containing a 2:1 ratio of cyclohexylammonium bromide and copper(II) bromide. A few drops of hydrobromic acid were added to the solution to avoid hydrolysis of the Cu(II) ion (Willett, 2004).

### Refinement

H atoms were positioned geometrically and refined using a riding model, with C—H = 0.97–0.98 Å, N—H = 0.89 Å and with *U*_iso_(H) = 1.2*U*_eq_(C) or *U*_iso_(H) = 1.5*U*_eq_(N).

### Crystal data

**Table:**

(C_6_H_14_N)_2_[CuBr_4_]	*F*(000) = 566
*M**_r_* = 583.51	*D*_x_ = 2.012 Mg m^−^^3^
Monoclinic, *P*2_1_/*c*	Mo *K*α radiation, λ = 0.71073 Å
Hall symbol: -P 2ybc	Cell parameters from 2203 reflections
*a* = 14.372 (3) Å	θ = 2.3–27.5°
*b* = 7.6483 (15) Å	µ = 9.42 mm^−^^1^
*c* = 9.1561 (18) Å	*T* = 293 K
β = 106.89 (3)°	Block, sepia
*V* = 963.0 (3) Å^3^	0.33 × 0.28 × 0.20 mm
*Z* = 2	

### Data collection

**Table:**

Rigaku SCXmini diffractometer	1468 reflections with *I* > 2σ(*I*)
Radiation source: fine-focus sealed tube	*R*_int_ = 0.092
Graphite monochromator	θ_max_ = 27.5°, θ_min_ = 3.1°
ω scans	*h* = −18→18
Absorption correction: multi-scan (*CrystalClear*; Rigaku, 2005)	*k* = −9→9
*T*_min_ = 0.056, *T*_max_ = 0.152	*l* = −11→11
9579 measured reflections	2 standard reflections every 150 reflections
2199 independent reflections	intensity decay: none

### Refinement

**Table:**

Refinement on *F*^2^	Primary atom site location: structure-invariant direct methods
Least-squares matrix: full	Secondary atom site location: difference Fourier map
*R*[*F*^2^ > 2σ(*F*^2^)] = 0.047	Hydrogen site location: inferred from neighbouring sites
*wR*(*F*^2^) = 0.095	H-atom parameters constrained
*S* = 1.03	*w* = 1/[σ^2^(*F*_o_^2^) + (0.024*P*)^2^] where *P* = (*F*_o_^2^ + 2*F*_c_^2^)/3
2199 reflections	(Δ/σ)_max_ = 0.001
88 parameters	Δρ_max_ = 0.62 e Å^−^^3^
0 restraints	Δρ_min_ = −0.77 e Å^−^^3^
0 constraints	

### Fractional atomic coordinates and isotropic or equivalent isotropic displacement parameters (Å^2^)

**Table:**

	*x*	*y*	*z*	*U*_iso_*/*U*_eq_	
Cu1	0.0000	0.0000	1.0000	0.0342 (2)	
Br2	0.17788 (4)	0.00282 (7)	1.09313 (6)	0.03796 (18)	
Br3	0.00212 (4)	0.25662 (7)	0.84636 (6)	0.04466 (19)	
N1	0.1634 (3)	0.9201 (6)	0.4590 (4)	0.0399 (11)	
H1E	0.1113	0.9760	0.4690	0.060*	
H1C	0.1627	0.8100	0.4898	0.060*	
H1D	0.1627	0.9216	0.3615	0.060*	
C1	0.4344 (4)	1.0010 (7)	0.6360 (6)	0.0484 (16)	
H1A	0.4396	1.1186	0.5994	0.058*	
H1B	0.4905	0.9351	0.6282	0.058*	
C2	0.4348 (5)	1.0087 (7)	0.8026 (6)	0.0525 (17)	
H2A	0.4376	0.8909	0.8429	0.063*	
H2B	0.4923	1.0709	0.8618	0.063*	
C3	0.3442 (4)	1.1006 (8)	0.8188 (6)	0.0497 (16)	
H3A	0.3442	1.0960	0.9247	0.060*	
H3B	0.3459	1.2225	0.7906	0.060*	
C4	0.2518 (4)	1.0177 (7)	0.7197 (5)	0.0389 (14)	
H4A	0.1961	1.0857	0.7260	0.047*	
H4B	0.2453	0.9006	0.7563	0.047*	
C5	0.2537 (4)	1.0092 (6)	0.5542 (5)	0.0309 (12)	
H5A	0.2542	1.1290	0.5166	0.037*	
C6	0.3421 (4)	0.9158 (8)	0.5364 (5)	0.0400 (14)	
H6A	0.3419	0.9197	0.4304	0.048*	
H6B	0.3403	0.7941	0.5654	0.048*	

### Atomic displacement parameters (Å^2^)

**Table:**

	*U*^11^	*U*^22^	*U*^33^	*U*^12^	*U*^13^	*U*^23^
Cu1	0.0304 (6)	0.0312 (5)	0.0383 (5)	−0.0027 (4)	0.0055 (4)	0.0048 (4)
Br2	0.0322 (4)	0.0389 (3)	0.0412 (3)	−0.0035 (3)	0.0082 (3)	0.0022 (2)
Br3	0.0428 (4)	0.0384 (3)	0.0463 (3)	−0.0101 (3)	0.0026 (3)	0.0103 (3)
N1	0.031 (3)	0.046 (3)	0.039 (2)	0.005 (2)	0.004 (2)	0.006 (2)
C1	0.035 (4)	0.066 (4)	0.044 (3)	0.004 (3)	0.011 (3)	0.008 (3)
C2	0.043 (4)	0.066 (4)	0.039 (3)	−0.001 (3)	−0.002 (3)	−0.001 (3)
C3	0.060 (5)	0.052 (4)	0.037 (3)	−0.008 (3)	0.012 (3)	−0.015 (3)
C4	0.030 (4)	0.048 (4)	0.036 (3)	0.004 (3)	0.006 (3)	−0.004 (3)
C5	0.030 (3)	0.029 (3)	0.031 (3)	−0.001 (3)	0.006 (2)	0.001 (2)
C6	0.032 (4)	0.058 (4)	0.032 (3)	0.002 (3)	0.013 (3)	−0.005 (3)

### Geometric parameters (Å, º)

**Table:**

Cu1—Br3^i^	2.4201 (6)	C2—H2A	0.9700
Cu1—Br3	2.4201 (6)	C2—H2B	0.9700
Cu1—Br2	2.4488 (9)	C3—C4	1.513 (7)
Cu1—Br2^i^	2.4488 (9)	C3—H3A	0.9700
N1—C5	1.500 (6)	C3—H3B	0.9700
N1—H1E	0.8900	C4—C5	1.525 (6)
N1—H1C	0.8900	C4—H4A	0.9700
N1—H1D	0.8900	C4—H4B	0.9700
C1—C6	1.521 (7)	C5—C6	1.507 (7)
C1—C2	1.525 (6)	C5—H5A	0.9800
C1—H1A	0.9700	C6—H6A	0.9700
C1—H1B	0.9700	C6—H6B	0.9700
C2—C3	1.525 (7)		
			
Br3^i^—Cu1—Br3	180.0	C4—C3—C2	112.0 (4)
Br3^i^—Cu1—Br2	89.59 (3)	C4—C3—H3A	109.2
Br3—Cu1—Br2	90.41 (3)	C2—C3—H3A	109.2
Br3^i^—Cu1—Br2^i^	90.41 (3)	C4—C3—H3B	109.2
Br3—Cu1—Br2^i^	89.59 (3)	C2—C3—H3B	109.2
Br2—Cu1—Br2^i^	180.000 (9)	H3A—C3—H3B	107.9
C5—N1—H1E	109.5	C3—C4—C5	110.3 (5)
C5—N1—H1C	109.5	C3—C4—H4A	109.6
H1E—N1—H1C	109.5	C5—C4—H4A	109.6
C5—N1—H1D	109.5	C3—C4—H4B	109.6
H1E—N1—H1D	109.5	C5—C4—H4B	109.6
H1C—N1—H1D	109.5	H4A—C4—H4B	108.1
C6—C1—C2	111.4 (5)	N1—C5—C6	109.6 (4)
C6—C1—H1A	109.3	N1—C5—C4	109.6 (4)
C2—C1—H1A	109.3	C6—C5—C4	112.8 (4)
C6—C1—H1B	109.3	N1—C5—H5A	108.3
C2—C1—H1B	109.3	C6—C5—H5A	108.3
H1A—C1—H1B	108.0	C4—C5—H5A	108.3
C3—C2—C1	111.1 (5)	C5—C6—C1	110.4 (4)
C3—C2—H2A	109.4	C5—C6—H6A	109.6
C1—C2—H2A	109.4	C1—C6—H6A	109.6
C3—C2—H2B	109.4	C5—C6—H6B	109.6
C1—C2—H2B	109.4	C1—C6—H6B	109.6
H2A—C2—H2B	108.0	H6A—C6—H6B	108.1

Symmetry code: (i) −*x*, −*y*, −*z*+2.

### Hydrogen-bond geometry (Å, º)

**Table:**

*D*—H···*A*	*D*—H	H···*A*	*D*···*A*	*D*—H···*A*
N1—H1*D*···Br2^ii^	0.89	2.60	3.474 (4)	166
N1—H1*C*···Br2^iii^	0.89	2.56	3.445 (4)	174
N1—H1*E*···Br3^iv^	0.89	2.62	3.341 (4)	138

Symmetry codes: (ii) *x*, *y*+1, *z*−1; (iii) *x*, −*y*+1/2, *z*−1/2; (iv) *x*, −*y*+3/2, *z*−1/2.
